# The Effect of Osmotic Dehydration Conditions on the Potassium Content in Beetroot (*Beta vulgaris* L.)

**DOI:** 10.3390/molecules29235509

**Published:** 2024-11-21

**Authors:** Bartosz Kulczyński, Joanna Suliburska, Anna Gramza-Michałowska, Andrzej Sidor, Przemysław Łukasz Kowalczewski, Anna Brzozowska

**Affiliations:** 1 Department of Gastronomy Science and Functional Foods, Faculty of Food Science and Nutrition, Poznań University of Life Sciences, Wojska Polskiego 31, 60-624 Poznań, Poland; anna.gramza@up.poznan.pl (A.G.-M.); andrzej.sidor@up.poznan.pl (A.S.); anna.brzozowska@up.poznan.pl (A.B.); 2 Department of Human Nutrition and Dietetics, Faculty of Food Science and Nutrition, Poznań University of Life Sciences, Wojska Polskiego 31, 60-624 Poznań, Poland; joanna.suliburska@up.poznan.pl; 3 Department of Food Technology of Plant Origin, Faculty of Food Science and Nutrition, Poznań University of Life Sciences, Wojska Polskiego 31, 60-624 Poznań, Poland; przemyslaw.kowalczewski@up.poznan.pl

**Keywords:** beetroot, food fortification, osmotic dehydration, potassium, inulin, erythritol

## Abstract

Osmotic dehydration as a process of removing water from food by immersing the raw material in a hypertonic solution is used primarily to extend the shelf life of products and as a pretreatment before further processing steps, such as drying and freezing. However, due to the bi-directional mass transfer that occurs during osmotic dehydration, the process can also be used to shape sensory properties and enrich the plant matrix with nutrients. The purpose of this study was to evaluate the effect of osmotic dehydration on the absorption of potassium by beet pulp immersed in various hypertonic solutions (sucrose, inulin, erythritol, xylitol solutions) with the addition of three chemical forms of potassium (gluconate, citrate, chloride) using variable process conditions. The study proved that osmotic dehydration is an effective way to enrich food. The highest potassium content (5779.03 mg/100 g) was found in a sample osmotically dehydrated in a 50% erythritol solution with 5.0% potassium chloride addition with a process that lasted 180 min and took place at 30 °C. The results obtained indicate the high potential of osmotic dehydration in improving the health values of food products. In addition, the antioxidant activity and proximate composition of osmotically dehydrated samples were also characterized in this study.

## 1. Introduction

Osmotic dehydration is a process in which water is removed from biological material, such as fruits, vegetables, or meat, by placing it in a solution with a high concentration of osmotically active substances. Due to the higher concentration of solutes in the solution, water from inside the cells migrates outward across the semi-permeable cell membrane, seeking to equalize the concentrations on both sides of the membrane. At the same time, some solutes in the solution can penetrate the cells, which can lead to changes in the taste, texture, physicochemical properties, or nutritional value of the dehydrated product. Usually, osmotic dehydration is used as a method of preliminary water removal before further drying processes, thus reducing the time and cost of these processes. In addition, this technique is associated with less degradation of nutrients, and as a result, the nutritional value and sensory qualities of the product are largely preserved. Of the osmotically active substances, sucrose and sodium chloride are the most commonly used [1,2]. However, due to the fact that during osmotic dehydration, these compounds migrate into the interior of the dehydrated material and that these substances are perceived as unfavorable for health, other osmotically active compounds, including inulin, oligofructose, and polyols such as sorbitol, xylitol, and erythritol, are increasingly used in research. In addition, beet molasses [3] and fruit juice concentrates, such as apple juice and cranberry juice, which, in addition to natural sugar, usually contain bioactive compounds, including polyphenols, are also sometimes used. Moreover, during osmotic dehydration, other compounds that have been dissolved in the solution can move from the hypertonic solution into the interior of the dehydrated plant matrix. This makes it possible to influence the sensory and nutritional characteristics of the dehydrated material by choosing the proper compounds and setting optimal process conditions [4]. For example, Kowalska et al. showed that osmotic dehydration had a favorable effect on the sensory properties of Japanese quince chips osmotically dehydrated in chokeberry and apple juice concentrates [5]. They showed high sensory rankings in terms of overall desirability (weight of color, taste, crispness, and flavor). The researchers proved that the use of drying methods with pre-osmotic treatment allows dried material to be obtained with sensory properties comparable to those obtained by freeze-drying. In addition, Giannakourou et al. showed that the osmotic dehydration of tomatoes in solutions of non-conventional sweeteners resulted in improved tomato texture [6]. As mentioned, osmotic dehydration may also shape the nutritional value of food products. For example, in our previous study, osmotic dehydration was shown to be an effective way to enrich pumpkin flesh with calcium when various chemical forms of calcium, including calcium carbonate, calcium chloride, and calcium lactate, were added to the hypertonic solution. It was observed that the calcium content of pumpkin dehydrated in a 50% inulin solution with calcium carbonate added at 5% at 50 °C for 2 h was 1328.4 mg/100 g. A similar effect was obtained under similar conditions when inulin was replaced by a solution of xylitol (50%)—the calcium content was 1380.4 mg/100 g. In contrast, a lower calcium level in the plant matrix was found when a 2.5% addition of calcium carbonate was used and when osmotic dehydration lasted for a shorter time (30 and 60 min). In these cases, the calcium content was about 3–4 times lower (426.3 and 356.4 mg/100 g, respectively) [7]. Moreover, it has been confirmed that dehydrated pumpkin pulp enriched in calcium can be used to produce foods with enhanced nutritional values. For example, a method of producing bread, vegetable paste, and soup using such an enriched product has been patented [8,9,10]. In addition, it should be emphasized that consumption of a dehydrated product enriched in specific nutrients is not only associated with the provision of higher amounts of such nutrients with the diet, which can counteract the occurrence of certain nutritional deficiencies, but it can additionally result in specific physiological effects. For example, a study showed that feeding pumpkin enriched in calcium lactate and calcium carbonate to rats increased calcium content in femurs, improved bone regeneration in rats after ovariectomy, and decreased the number of femoral bone marrow adipocytes, as well as lowered serum leptin levels [11,12,13]. As mentioned, the effectiveness of osmotic dehydration in enriching plant tissue with nutrients is influenced by process conditions. The literature, as well as our own research, indicates that the transfer of components is influenced by factors such as the type of plant tissue, the type of osmotically active agents, the concentration of the osmotic solution, the time and temperature of the process, the ratio of the weight of the plant matrix to the weight of the solution, the type and concentration of the substance additionally added to the solution, as well as the size and shape of the dehydrated product. In our study, we observed a significant effect due to the concentration of calcium added, as well as the time of the process, while the type of osmotic substance and temperature were less important [7]. Considering all this information, it should be pointed out that osmotic dehydration is a promising process for influencing the nutritional value of food, and, therefore, further research on this subject is warranted. To date, many research results on the osmotic dehydration of various vegetables and fruits have been published in the scientific literature. Apples [14], pineapple [15], kumquat [16], peaches [17], strawberries [18], papaya [19], kiwi [20], mango [21], banana [22], sweet potatoes [3], pumpkin [23], carrots [24], and onions [25], among others, have been subjected to the mentioned process. However, the vast majority of the experiments were focused on studying the effects of osmotic dehydration on the kinetics of solids gain and water loss, as well as on the change in color, texture, and antioxidant content (including anthocyanins, ascorbic acid, carotenoids, vitamin E) of the dehydrated product [17,26]. The analysis of weight gain and water loss in the raw material during osmotic dehydration is a key step that affects the drying efficiency of raw materials. Osmotic dehydration reduces the water content of the raw material, which leads to a lower initial moisture content and results in a shorter drying time. In addition, sugars or salts that act as natural preservatives penetrate the raw material during osmosis, which prevents the growth of microorganisms during lower-temperature drying. Thus, the research published to date focuses more on the strictly technological aspect, rather than the nutritional (dietary) aspect. The results obtained in these experiments prove that osmotic dehydration is a highly effective method for mass exchange between the raw material and the hypertonic solution. This means that osmotic dehydration can be used to shape the nutritional value of products, including the production of foods enriched with certain nutrients. However, to date, there are not many studies that have analyzed the effect of adding nutritional compounds to a hypertonic solution on the nutritional value of a plant matrix undergoing osmotic dehydration. Such a study was conducted by Chardonnet et al., who observed an increase in the calcium content of apple slices osmotically dehydrated in sucrose solutions with the addition of calcium chloride. The calcium content of the osmotically dehydrated apple slices was 40 times higher than in the control sample [27]. In turn, Emser et al. confirmed the usefulness of osmotic dehydration for enriching apples with probiotic bacteria (*Lactobacillus plantarum*) [28]. In another experiment, osmotic dehydration was used to enrich pear in calcium and zinc using the addition of calcium lactate and zinc acetate [29]. The promising results of these studies, and the small number of them, prove that it is worth continuing work on evaluating the impact of osmotic dehydration on the nutritional value of the final product. One of the plant raw materials that can be subjected to osmotic dehydration is beet. To date, only a few studies have been conducted using this root vegetable in this field. However, these studies have not involved enriching it with selected nutrients [30,31,32]. Beet is an important source of many nutrients, including dietary fiber (2.8 g/100 g), potassium (325 mg/100 g), folate (109 µg/100 g), and betaine (129 mg/100 g) [33], as well as providing betacyanins, betaxanthins [34], and inorganic nitrogen compounds, which significantly determine its health properties, including its beneficial effects on the cardiovascular system [35]. Despite the fact that beets are listed as a source of potassium [36], it should be noted that compared to human requirements for this mineral, the potassium content is not very high. Taking into account the fact that in one beet (about 100 g) there is 325 mg of potassium, such a portion meets the daily requirement for potassium (3500–4700 mg) in only about 7–9% of people [37]. Of course, there are many more potassium-containing products in the human diet. However, it should be noted that the scientific literature clearly indicates that the average intake of potassium in the world is only 2250 mg/day, and only 14% of the population meets the recommended level of intake of this nutrient [38]. At the same time, it is worth noting that dietary potassium deficiency is associated with a number of health complications. Experiments have shown that low potassium intake and low serum potassium levels are associated with a higher risk of atrial fibrillation [39], a higher incidence of type 2 diabetes [40], and a higher risk of kidney stone formation [41], as well as a higher risk of mortality in patients with cardiovascular diseases [42]. In view of this information, it seems reasonable to develop potassium-enriched foods to prevent potassium deficiency and related health problems. As shown, there are strong indications suggesting that osmotic dehydration may be an effective way to increase potassium levels in plant products, including beets. Therefore, within the framework of this study, osmotic dehydration was carried out to enrich beet pulp with this element, while establishing the most optimal process conditions. Nevertheless, it should be noted that osmotic dehydration reduced the antioxidant activity of beet pulp; therefore, the next step may be to optimize the process not only in terms of the efficiency of osmotic dehydration in mineral enrichment but also in terms of increased antioxidant retention.

## 2. Results and Discussion

### 2.1. Potassium Content in Dehydrated Beetroot Flesh (First Stage)

In the first stage of the study, the effect of the osmotic substance and chemical form of potassium and its concentration on the potassium content of beet pulp was analyzed. It was shown that the highest potassium content was characterized by samples dehydrated with potassium chloride (Table 1). A statistically significant (*p* < 0.05) higher level of potassium was observed for all analyzed samples compared to samples dehydrated with potassium citrate and potassium gluconate. This fact is due not only to the high solubility of potassium chloride in water [43] but also probably primarily to the high percentage of potassium in this molecule (~52.5%). As shown in our own earlier study [7], the percentage of the mineral is more important for the effect achieved than its solubility in water. Indeed, it was shown that the highest levels of calcium in dehydrated pumpkin were characterized by samples dehydrated with the addition of water-insoluble calcium carbonate (compared to samples dehydrated with the addition of calcium lactate and calcium chloride). At the same time, it should be noted that the statistically significant lowest (*p* < 0.05) potassium content was characterized by samples dehydrated with potassium gluconate, which among the analyzed chemical forms of potassium contains the lowest percentage of potassium (~16.7%). Analyzing the effect of osmotic substance on potassium content, it can be noted that in terms of potassium chloride, the highest amounts of potassium were found in samples dehydrated in solutions of inulin, xylitol, and erythritol. Statistically significant lower (*p* < 0.05) potassium content was found in samples dehydrated in sucrose solutions and samples that were placed in water. The results of the multivariate analysis of variance (Table 2) showed that the type of osmotically active substance and the chemical form of potassium had almost the same effect on the obtained effect (*η*^2^: 0.99 vs. 1.00). At the same time, it should be noted that a higher potassium content was found in samples dehydrated in solutions of higher concentration (50 vs. 25%). Of all the samples examined, the highest potassium content was found in samples dehydrated in 50% erythritol solution (5165.99 mg/100 g), 50% inulin solution (5059.92 mg/100 g), and 50% xylitol solution (5007.30 mg/100 g). At the same time, no statistically significant differences were found between these samples. In contrast, the lowest potassium content was found in samples that were placed in water with potassium gluconate (744.32 mg/100 g). Potassium content was analyzed not only in beets subjected to osmotic dehydration alone but also in samples subjected to osmotic dehydration that were freeze-dried. The potassium content of these samples is shown in Table 3. The highest potassium content was in samples dehydrated in a 25% solution of erythritol with potassium chloride (16,497.01 mg/100 g) and samples immersed in water with potassium chloride (16,046.25 mg/100 g). It should be noted that the level of potassium in samples that were additionally freeze-dried is determined by the dry matter content.

### 2.2. Potassium Content in Dehydrated Beetroot Flesh (Second Stage)

Based on the results obtained in the first stage, a second stage of the study was carried out to analyze the effect of other factors (time and temperature of the process, concentration of the chemical form of potassium) on potassium content (Table 4). The samples characterized by the highest potassium content were selected for the second stage. These were samples dehydrated in 50% solutions of inulin and erythritol with the addition of potassium chloride. It was shown that the result was significantly influenced by the concentration of the added potassium compound. For all samples, a statistically significant (*p* < 0.05) higher potassium content was observed in beets dehydrated with 5% potassium chloride addition, compared to beets dehydrated with 2.5% potassium chloride addition. At the same time, it was found that the efficiency of osmotic dehydration as a method of enriching the plant matrix with potassium was significantly affected by the time of the process. It was shown that with an increase in the time of osmotic dehydration, the increase in potassium levels in the samples studied was higher. Statistically significant differences (*p* < 0.05) were found for all samples. In addition, it was shown that process temperature (50 °C vs. 30 °C ) was also a factor determining potassium levels in the dehydrated material. In most cases (eight pairs of samples compared), there was a statistically significantly higher potassium content in samples dehydrated at 50 °C. Only in two pairs were statistically significantly higher potassium levels observed in beets dehydrated at 30 °C (inulin solution with 2.5% potassium chloride addition, 180 min; erythritol solution with 5.0% potassium chloride addition, 180 min). The conducted research showed that the highest potassium content was characterized by samples osmotically dehydrated under the following conditions: (1) 50% erythritol solution with 5.0% potassium chloride addition, 30 °C, 180 min (5779.03 mg/100 g), (2) 50% inulin solution with 5.0% potassium chloride addition, 30 °C, 180 min (5457.28 mg/100 g), and (3) 50% erythritol solution with 5.0% potassium chloride addition, 50 °C, 180 min (5353.55 mg/100 g). The results indicate that it would be sufficient to consume ~25 g of dehydrated beet pulp enriched in potassium to cover the recommended daily intake of potassium (3500–4700 mg) [44] in 30–40% of people. This fact supports the usefulness of osmotic dehydration for enriching the plant matrix with minerals deficient in the human diet. Conversely, the lowest potassium content (<3000 mg/100 g) was found in samples dehydrated with a 2.5% addition of potassium chloride: (1) 50% erythritol solution, 60 min, 30 °C (2477.18 mg/100 g), (2) 50% inulin solution, 60 min, 30 °C (2681.04 mg/100 g), and (3) 50% erythritol solution, 60 min, 50 °C (2794.83 mg/100 g). Multivariate variance analysis (Table 5) confirmed that among the factors studied (type of osmotically active substance, concentration of potassium addition, time, temperature), the level of potassium added (*η*^2^ = 0.99) and process time (*η*^2^ = 0.99) had the highest impact on the result. Conversely, the lowest significance was found for process temperature (*η*^2^ = 0.54). Considering beet pulp that was freeze-dried after osmotic dehydration, the highest potassium content (>10,000 mg/100 g) was found in five samples: (1) 50% erythritol solution with 5.0% potassium chloride addition, 180 min, 30° (12,078.18 mg/100 g), (2) 50% inulin solution with 5.0% potassium chloride addition, 180 min, 50° (11,950.84 mg/100 g), (3) 50% erythritol solution with 5.0% potassium chloride addition, 120 min, 50° (11,008.40 mg/100 g), (4) 50% inulin solution with 5.0% potassium chloride addition, 120 min, 50° (10,742.22 mg/100 g), and (5) 50% erythritol solution with 5.0% potassium chloride addition, 180 min, 50° (10,546.49 mg/100 g) (Table 6). It should be noted that potassium belongs to the family of thermostable components, hence freeze-drying has no significant effect on the loss of these components, as confirmed by other studies [45]. At the same time, it should be noted that the higher potassium content in freeze-dried samples is due to the removal of water from the material, resulting in an increased concentration of the components it contains. A similar principle applies to osmotically dehydrated samples in solutions with a lower concentration of the osmotically active substance. Based on the results obtained, the level of potassium absorption by beet pulp after osmotic dehydration was also determined (Table 7). It was observed that most of the samples dehydrated in 50% inulin solution (10 out of 12 pairs) had a higher % absorption level compared to beets dehydrated in 50% erythritol solution. In addition, a higher % absorption level was noted for all samples analyzed when 2.5% potassium chloride was added to the hypertonic solution, compared to samples with 5.0% potassium chloride addition. At the same time, a higher absorption level was found in samples dehydrated for a longer time (120 and 180 min). In comparison with the amount of potassium added to the hypertonic solution, the statistically significant highest level of potassium absorption (69.56%) was recorded in beets osmotically dehydrated in 50% inulin solution with 2.5% potassium chloride addition at 30 °C for 120 min. Conversely, the lowest results (26.29 and 26.19%) were obtained in samples dehydrated in 50% erythritol solution with 5.0% potassium chloride addition at 30 and 50 °C, when the process lasted 60 min. The concentration of the osmotic substance (e.g., sucrose, inulin, etc.) has the greatest influence on mass transfer between the raw material and the hypertonic solution. In contrast, as shown, the increase in potassium levels in osmotically dehydrated samples is not necessarily proportional to the concentration of added potassium compounds. The analyses carried out showed that samples placed in a solution with a higher concentration (5.0 vs. 2.5%) are characterized by a higher potassium content, but in relation to the total potassium content of the solution, the absorption efficiency is lower, which may be due to some limitations related to the high increment of the osmotically active substance. To date, relatively few studies have been published that focus on the use of osmotic dehydration to enrich the plant matrix with selected nutrients. For example, several studies have analyzed the effects of calcium compounds added to an osmotic solution on the structural integrity of the cell walls of the dehydrated raw material, as well as on impregnation, kinetics of water loss, dry weight gain, or water activity. For example, Silva et al. used the addition of calcium lactate at concentrations of 2 and 4% during osmotic dehydration of pineapple using 40 and 50% sucrose solutions. Similar to the present study, they observed that samples dehydrated in solutions with higher calcium levels (4%) had higher calcium content, compared to samples that were dehydrated with calcium addition at 2%. In addition, they showed that there was a higher calcium increment with increasing dehydration time. Samples dehydrated for 6 h had a calcium content of ~90 mg/100 g. With mixed results between samples dehydrated in 40 and 50% sucrose solutions. Samples dehydrated for 1, 2, and 4 h had higher calcium levels when 50% sucrose solution was used, which is also consistent with our observations that calcium absorption levels are affected by the concentration of the osmotic solution [46]. Similarly, Germer et al. analyzed the effect of calcium addition on structural changes and mechanical properties of papaya pieces after osmotic dehydration. They used the following process conditions: sucrose solution (40 and 60° Brix), calcium lactate addition (5, 10, 15, 20 g/kg), 30 °C, and agitation (120 rpm) for 2 h. The researchers showed that the level of calcium addition had a significant effect on the calcium content of osmotically dehydrated melon. The highest calcium content (about 4000 mg/kg) was observed in the dehydration sample with the addition of calcium lactate at 20 g/kg [47]. In contrast, Rodríguez-Ramírez confirmed that calcium gain is affected by osmotic dehydration time. They showed the highest calcium content in chilacayote squash dehydrated with Ca(OH)_2_ for 4 h (vs. 3.0 and 1.5 h). Moreover, they showed that the temperature of the process (20, 35, and 50 °C) had only a slight effect on calcium levels, which is consistent with the results obtained in our experiment [48]. It should be noted that the titration method (titration of calcium with potassium manganate (KMnO_4_)) was used to determine the mineral content in the cited experiment. This involves the oxidation of calcium ions in the presence of oxalate, forming calcium oxalate, which is then titrated with a solution of potassium manganate. This method appears to be less sensitive, accurate, and reproducible compared to the atomic absorption spectrometry (ASA) that we used. In another study, Pereira et al. subjected guava to osmotic dehydration using 60 °Brix sucrose solution (pH 6.7) with calcium chloride or calcium lactate (5, 10, 15, 20, 25 g/kg). They confirmed that the calcium content of the dehydrated material was significantly influenced by the chemical form of calcium. They showed that higher calcium content was characterized by samples with calcium chloride addition. The higher the calcium addition used, the higher the calcium content of the dehydrated guava. With calcium chloride (25 g/kg) applied, the sample had a calcium content of ~8000 mg/kg. In contrast, in the sample dehydrated with calcium lactate (25 g/kg), the calcium content was ~4500 mg/kg [49]. In this study, the inductively coupled plasma atomic emission spectrometry (ICP-AES) method was used to determine calcium. This technique involves the excitation of the element’s atoms in a sample using a very high-temperature plasma, resulting in the emission of light at characteristic wavelengths, which allows precise determination of mineral content. The addition of calcium during osmotic dehydration was also conducted by Mauro et al. The researchers subjected apples to osmotic dehydration using a 40% sucrose solution with calcium lactate (4.0%). They found a significant increase in the calcium content of the dehydrated samples. They showed that the calcium levels in the apples increased with increasing process time. The highest result was obtained in apples dehydrated simultaneously with ascorbic acid for 240 min (195.20 mg/100 g). In comparison, in samples dehydrated for 120, 60, and 30 min, the calcium content was respectively: 140.01, 108.57, and 81.05 mg/100 g. In contrast, the calcium content of the samples that did not undergo osmotic dehydration was 2.78 mg/100 g [50]. Based on published research results on the effect of the addition of calcium compounds on the calcium content of plant materials subjected to osmotic dehydration, we conducted an experiment. This consisted of using osmotic dehydration to enrich pumpkin flesh with calcium. Unlike many other studies, this experiment was designed specifically to enrich pumpkin in this element. Based on our study, we confirmed that the calcium content is significantly influenced by the concentration of calcium addition and the process time. We observed the highest calcium content in samples dehydrated in 50% inulin and xylitol solutions with 5% calcium carbonate addition for 2 h (5% calcium carbonate (12.5 g) refers to a weight-based addition to all osmotic solutions (250 g)). The calcium content of these samples was 1409.0 and 1492.9 mg/100 g, respectively [7]. Based on the results, we designed a further study to enrich beet pulp with potassium and magnesium (data not yet published). There is a lack of research that has analyzed the addition of minerals other than calcium as a possibility for food enrichment by osmotic dehydration. However, Zhao and Park conducted a study in which they investigated vitamin E and zinc content following vacuum impregnation of apples. In their study, they used a 0.04% addition of zinc lactate. After treatment, they observed a statistically significant increase in the zinc content of the apple slices (1.7 mg/100 g), representing 17% of the daily requirement for this element. Thus, the authors of the experiment confirmed that the use of hypertonic solutions with the addition of other components (including minerals) is an effective method to enrich plant tissue with these compounds [51]. Similar conclusions were obtained by Nagai et al., who used the addition of ascorbic acid during the osmotic dehydration of mangoes. The vitamin C content of the starting raw material was 40.28 mg/100 g. Osmotic dehydration resulted in a vitamin C content of up to 400.53 mg/100 g, which means that up to a 10-fold increase in the content of this nutrient was achieved [52]. An interesting study was also conducted by Vijay et al., who demonstrated that osmotic dehydration can also be used to enrich foods with probiotic bacteria. They showed that osmotic dehydration of pineapple in 40 and 50 °Brix sucrose solutions with the addition of probiotic bacteria led to a significant increase in their levels in the dehydrated material. The content of *Lactiplantibacillus plantarum* was 7.30–8.04 log CFU/g, while *Lacticaseibacillus casei* was 7.69–8.27 log CFU/g. Furthermore, it is noteworthy that high probiotic viability was obtained during gastrointestinal stress and storage [53]. All these data confirm that osmotic dehydration can be successfully used to design the nutritional value of food products.

### 2.3. Antioxidant Activity and Proximate Composition of Dehydrated Beetroot

In order to characterize the nutritional value of osmotically dehydrated beet, their antioxidant activity was determined. Tests using DPPH radicals showed that fresh beet and freeze-dried beet, which had not undergone osmotic dehydration, had the highest antioxidant potential. It was confirmed that osmotic dehydration has an adverse effect on antioxidant activity (Table 8). Osmotically dehydrated samples (fresh and freeze-dried) showed statistically significantly lower DPPH radical scavenging capacity by 54.65–68.75%. Furthermore, it was shown that samples osmotically dehydrated at a higher temperature (50 vs. 30 °C) had lower antioxidant activity. A similar effect was also observed in the ABTS radical scavenging assay. Osmotic dehydration was shown to reduce ABTS cation radical scavenging efficiency by 41.73–66.12%. In contrast, the most significant decrease in antioxidant activity was observed in the oxygen radical absorbance capacity (ORAC) test. A reduction in the ability of antioxidants to protect the fluorescent probe from free radical damage by 85.23–89.45% was recorded. This effect is due to the loss of antioxidant compounds present in beetroot. Constituents such as betacyanins, ascorbic acids, phenolic acids, and flavonoids are water-soluble and/or thermolabile. For example, Sawicki et al. showed that boiling red beetroot reduces betalains by 51–61% [54]. It is important to optimize the process to obtain the highest retention of bioactive compounds in the osmotically dehydrated beet. The addition of ingredients with high antioxidant activity, including, for example, spices, to the hypertonic solution may also be a considered solution. The effect of the addition of spices on the antioxidant potential of osmotically dehydrated beet is the subject of further research. In contrast to the enrichment of foods with selected nutrients by osmotic dehydration, a number of studies have been published in the literature analyzing the effect of this process on the antioxidant potential of dehydrated samples. Udomkun et al. have demonstrated that osmotic dehydration of papaya in hypertonic sucrose solution (30 °Brix) resulted in a reduction of bioactive compounds with antioxidant properties. Compared to fresh material, there was a statistically significant reduction in beta-carotene (1.71 vs. 0.53 mg/100 g), beta-cryptoxanthin (1.14 vs. 0.49 mg/100 g), and total lycopene (22.13 vs. 12.31 mg/100 g). As a result, there was a reduction in DPPH radical scavenging activity (7.3 vs. 6.3 mM TE/g), ABTS radical cation scavenging activity (5.6 vs. 5.2 mM TE/g), and FRAP capacity (6.4 vs. 6.0 mM TE/g). This effect was probably due to mass transfer, as some of the compounds mentioned have relatively good solubility in water. In addition, carotenoids are photo-, oxygen-, and thermo-labile compounds, hence prolonged osmotic dehydration (6 h) promoted the degradation of these compounds [55]. A similar result was reported by Rahman et al., who confirmed a reduction in the antioxidant activity of nutmeg pericarp following osmotic dehydration in aqueous sugar solution (60, 70, 80%). The researchers showed that there was an increasingly significant reduction in total phenolic content as the process was prolonged (3, 6, 9, 12, 15 h). At the same time, there was a statistically significant reduction in free radical scavenging activities (DPPH assay). It should be noted that, of the osmotically dehydrated samples, nutmeg pericarps placed in 80% sugar solution had the highest antioxidant activity, indicating that the concentration of the osmotic solution can affect the retention of bioactive compounds [56]. A reduction in antioxidant content and antioxidant activity was also reported by Wiktor et al. They showed that osmotic dehydration of strawberries at 30 for 3 h in different solutions (sucrose: 50%; mannitol: 20% and 30%; sorbitol: 20, 30, and 40%) led to a statistically significant reduction in the ability of strawberries to scavenge DPPH and ABTS radicals. The researchers showed that this effect was associated with a reduction in total anthocyanin and vitamin C content. Despite the low temperature, the long process time may have negatively affected the loss of bioactive compounds [57]. Devic et al. also confirmed a reduction in the concentration of bioactive compounds during the osmotic dehydration of apples. They showed that the loss of these compounds was significantly influenced by the time and temperature of the process. With increasing osmotic dehydration time (30, 60, 90, 120, 150, 180 min), there was a more significant reduction in proanthocyanidins, monomeric catechins, and hydroxycinnamic acids. Furthermore, also an increase in process temperature (60 vs. 45 °C) had a negative effect on the retention of bioactive compounds. The most unfavorable result was found for monomeric catechins and hydroxycinnamic acids (loss of ~80%) during osmotic dehydration at 60 °C for 180 min [58]. A contrary observation was reported by Osae et al., who subjected ginger slices to osmotic dehydration. The authors showed a statistically significant increase in antioxidant activity (in DPPH, ABTS, FRAP, and CUPRAC tests) after osmotic dehydration of the raw material in a 20% sucrose solution at 30 °C for 30 min. Osae et al. suggested that this effect may be due to an increase in the extraction of bioactive compounds after the osmotic dehydration process. It should be noted that the authors conducted the experiment under mild conditions (30 °C/30 min), which may not have had a negative effect on the content of antioxidant compounds [59]. Interesting observations were made by Azeez et al., who demonstrated that osmotic dehydration can be a beneficial processing method to prevent excessive loss of bioactive compounds during further drying. In their experiment, tomato slices were osmotically dehydrated in 10% saline solution and the raw material was then subjected to oven drying. The researchers demonstrated that samples subjected to osmotic dehydration pre-treatment and then oven drying had higher flavonoid, phenolic, lycopene, and beta-carotene content compared to samples that had not been osmotically dehydrated previously. As a result, tomato slices subjected to osmotic dehydration were characterized by higher antioxidant activity, compared to untreated samples [60]. Similar conclusions were drawn by Islam et al., who demonstrated that hot-air dried papaya previously subjected to osmotic dehydration was characterized by a lower loss of total phenolic and ascorbic acids. The higher the osmotic solution concentration (60 vs. 50 vs. 40%), the higher the retention of bioactive compounds, confirming the observations made by Rahman et al. that solution concentration is an important determinant of the content of bioactive components in the dehydrated material. The authors showed that as the process temperature increased (35, 45, 55 °C), the antioxidant activity (% DPPH inhibition) of the dehydrated samples decreased [61]. An increase in the content of phenols (apigenin, luteolin, kaempferol, chrysin, *p*-coumaric acid, chlorogenic acid, gallic acid, and ferulic acid) and antioxidant activity was in turn confirmed in a study by Nićetin et al., who subjected celery root to osmotic dehydration. However, this effect was due to the fact that the raw material was placed in sugarbeet molasses, which was characterized by a high content of these compounds, demonstrating that by selecting an appropriate hypertonic solution, food can be modulated/enriched with components with antioxidant properties. As a result, the authors noted an increase in the activity of osmotically dehydrated celery root in the following tests: ABTS, DPPH, and FRAP [62]. Similar findings were obtained by Lech et al., who confirmed that osmotic dehydration of carrots and courgettes in chokeberry juice resulted in a significant increase in total polyphenol content and antioxidant activity in ABTS and FRAP assays [63]. Macronutrient content was also determined in the osmotically dehydrated samples. It was shown that the osmotically dehydrated beet was characterized by a lower content of protein, fat, and fiber, due to the transfer of these components from the plant tissue to the hypertonic solution by bidirectional mass exchange. With regard to macronutrients, the partial loss of them is not problematic, as beets are not an important source of them in the diet. However, it is unfavorable to reduce the antioxidant activity of beets, which are rich in antioxidants. Therefore, it was planned to optimize osmotic dehydration conditions for the highest possible antioxidant retention/preservation of antioxidant activity in subsequent studies. One of the solutions is the addition of spices during osmotic dehydration, which are likely to benefit the antioxidant potential of the dehydrated product. Nevertheless, despite the reduction in antioxidant activity, osmotically dehydrated beets can still be an important part of the diet for people with low potassium intake.

### 2.4. Texture Profile Analysis (TPA)

The study confirmed that osmotic dehydration has a significant effect on texture parameters (Table 9). It was shown that fresh beet and beet placed in water without the addition of potassium chloride and osmotically active substance had significantly the highest hardness (275.72 N). Osmotic dehydration resulted in a decrease in hardness, with the lowest value of this parameter (28.64 N) found for beet subjected to osmotic dehydration in 50% inulin solution and 5.0% potassium chloride. Also in previous studies [7], the highest hardness was observed for fresh pumpkin, while the lowest value was observed for pumpkin placed in a hypertonic solution (50% inulin) with the addition of calcium carbonate (5%). At the same time, there were no statistically significant differences between samples in terms of adhesiveness (*p* > 0.05), as well as springiness, except for beet subjected to osmotic dehydration with inulin solution and potassium chloride, which achieved a significantly higher result (0.68). In addition, it was observed that osmotic dehydration caused a significant reduction in gumminess and chewiness. At the same time, it was shown that samples placed in inulin solution (S4 and S7) had two times higher cohesiveness than samples placed in erythritol solution (S3 and S6). Such an effect may be due to the fact that inulin has a higher viscosity and higher water-binding capacity. The effect of osmotic dehydration on the texture of raw materials was also confirmed by Khuwijitjaru et al. [21]. The authors showed that the process improved the texture of mangoes (increase in chewiness and gumminess) that were subjected to hot air drying after osmotic dehydration. The contrary effect was noted in our study, which is probably due to the fact that in this experiment, non-dried material was used. In another experiment, Giannakourou et al. confirmed that the texture profile was affected by the type of osmotically active substance used [6]. For example, they showed that the highest chewiness and hardness were characterized by tomatoes dehydrated in a mixture of sucrose and oligofructose (1:1), while the highest elasticity was found in samples dehydrated in a mixture of isomaltulose/steviol glucosides + oligofructose and in a mixture of polydextrose/sucralose/steviol glucosides + oligofructose. The effect of the osmotically active substance on texture profile analysis was also observed in our study. For example, it was shown that beet dehydrated in erythritol solution (S6) showed higher hardness, gumminess, and chewiness than the sample dehydrated in inulin solution (S7).

## 3. Materials and Methods

### 3.1. Sample Collection

The material for the study was beetroot (*Beta vulgaris* L.) pulp, which was purchased from the ‘Mogilnica Valley’ Organic Farming Product Cooperative. Directly after purchase, the beetroot was peeled and cut into cubes sized 2.0 × 2.0 × 2.0 cm. Next, 50 ± 0.5 g of beet cubes were weighed and packed into separate boxes. These were then frozen at −80 °C until osmotic dehydration and further analysis.

### 3.2. Chemicals and Reagents

Nitric acid (65% and lanthanum chloride, 2,2-diphenyl-1-picrylhydrazyl, trolox, 2,2-azino-bis-(3-ethyl-benzthiazoline-6-sulfonic acid), potassium persulfate, dipotassium hydrogen phosphate anhydrous, sodium dihydrogen phosphate, fluorescein, 2,2′-azo-bis-2-amidinopropane dihydrochloride, methyl alcohol were purchased from Sigma-Merck (Poznan, Poland). Inulin (Orafti HSI), xylitol, and erythritol were purchased from HORTIMEX Sp. z o.o (Konin, Poland). Sucrose was purchased at a local market. Potassium gluconate, potassium citrate, and potassium chloride (NOW Foods, Goczałkowice-Zdrój, Poland) were purchased from an online store. The remaining reagents used for the analyses were purchased from AlfaChem (Lublin, Poland).

### 3.3. Osmotic Dehydration Procedure

The effect of different osmotic dehydration conditions on the potassium content of the samples was evaluated in two stages. The first stage analyzed the effect of the chemical form of potassium (potassium gluconate, potassium citrate, potassium chloride) and osmotically active substances and their concentrations (water, inulin solution 25 and 50%, xylitol solution 25 and 50%, erythritol solution 25 and 50%, sucrose solution 25 and 50%). It should be noted that the chemical forms of potassium used are approved for food use in accordance with Regulation No. 1925/2006 of the European Parliament and Council [64]. In addition, the selected chemical forms of potassium have different water solubility, as well as different elemental potassium content. Moreover, the organic forms of potassium (potassium citrate and potassium gluconate) and the inorganic form (potassium chloride) were selected for the study. Inulin, erythritol, and xylitol were chosen as osmotic actives as healthier alternatives to sucrose, which is one of the most commonly used components in studies on osmotic dehydration. Compared to sucrose, these ingredients have a lower energy value, do not significantly elevate blood glucose levels, do not cause dental caries, and have broad beneficial effects on health, including prebiotic properties, lowering triglycerides, anti-inflammatory properties, and reducing energy intake, among others [65,66,67,68,69]. The use of these osmotically active substances seems reasonable due to the adverse health effects of sucrose, including an increased risk of metabolic diseases [70], the prevalence of depression [71], or periodontal diseases [72]. Solutions of osmotically active substances (25%) were prepared by mixing 62.5 g of inulin, xylitol, erythritol, or sucrose with 187.5 g of water. For 50% solutions, 125 g of osmotically active substances and 125 g of water were used. Potassium compounds (12.5 g) were added to the prepared solutions placed in sealed glass vessels, which accounted for 5.0% of the weight of the solution. After carefully mixing the ingredients, previously frozen and weighed beet cubes (50 g) were placed in glass vessels (the ratio of raw plant material to the weight of the osmotically active substance solution was 1:5). The glass vessels were then capped and placed in a shaking water bath (SWB 22N) at 50 °C. The osmotic dehydration process was carried out for 120 min. After the process, the beets were drained in a sieve, then weighed and placed in the freezer (−80 °C) for 24 h. After this time, the samples were freeze-dried and weighed again. Freeze-drying (Alpha 1–2, Martin Christ) was carried out under the following conditions: ice condenser temperature, −55 °C, shelf temperature, +19 °C, 48 h. The cubes were then ground into powder using a grinder (Bosch, Berlin, Germany). Such prepared samples were used for further analysis, including determination of potassium content. The second stage of osmotic dehydration involved analyzing the effects of time and temperature of osmotic dehydration and the level of addition of potassium compounds. For this purpose, two temperature variants were used, namely, 30 and 50 °C s, and 3 time variants, namely, 60, 120, and 180 min. Based on the results obtained in the first stage of the study, samples dehydrated in 50% solutions of inulin and erythritol were selected. In addition, potassium chloride was selected from among the chemical forms of potassium. The samples dehydrated under these conditions had the highest potassium content. Moreover, both inulin and erythritol are compounds that, like potassium, have been shown to have positive effects on the cardiovascular system, including consumption being associated with reduced blood pressure [73], reduced risk of developing hypertension by 21% [74], reduced low-density lipoprotein (LDL) and triglyceride levels [75], and improved small vessel endothelial function, and reduced central aortic stiffness [76]. In order to compare the effect of the level of addition of the chemical form of potassium, 2.5 or 5.0% addition of potassium chloride was used. Sample preparation for the second stage of osmotic dehydration was similar to that used in the first stage. It should be noted that the selection of osmotic dehydration parameters for both the first and second stages was determined based on the results achieved in our previous studies and on a review of other research studies [7,14,77,78].

### 3.4. Determination of Potassium Content

After osmotic dehydration, the samples were freeze-dried and then ground into powdered form. Then, 1 g of sample was weighed into quartz crucibles and placed in a muffle furnace at 450 °C until complete mineralization. The samples were then dissolved using 1 mol/L nitric acid (Suprapure, Merck, Poznań, Poland). Analysis of the samples was conducted in triplicate. The mineral solutions were measured by flame atomic absorption spectrometry (ZA3000; Hitachi, Tokyo, Japan) [79]. The accuracy of the method used for determination was 96% for potassium. Detection limit: 3.0 ppb.

### 3.5. Extract Preparation for Antioxidant Activity Analysis

To determine the antioxidant activity of dehydrated beet pulp, sample extraction was carried out. Extraction conditions were based on previous studies [80,81,82]. A total of 5 g of lyophilized, osmotically dehydrated beet was weighed and dissolved in 50 mL (1:10 ratio) of methanol–water solution (80%). The prepared sample was transferred to flasks and placed in a shaking water bath (SWB 22N, Laboplay, Bytom, Poland) for 120 min at 50 °C. After extraction, the samples were centrifuged (1500 rpm, 10 min), filtered through blotting paper, and used for further analysis.

### 3.6. Antioxidant Activity Analysis

Antioxidant activity was analyzed using the DPPH radical scavenging activity assay, ABTS radical scavenging assay, and oxygen radical absorbance capacity (ORAC) assay.

Determination of antioxidant activity using DPPH radical was carried out according to the method developed by Brand-Williams et al. (1995) [83]. 0.01 g of DPPH was weighed and then transferred to a volumetric flask (25 mL) with solvent (80% methanol–water solution). To determine the antioxidant activity, 100 µL of extract was taken and 250 µL of DPPH reagent and 2.0 mL of solvent were added. The whole mixture was shaken with vortexing at ambient temperature (22 °C) and left in the dark for 20 min. The absorbance of the samples was measured at λ = 517 nm (SP 830, Metertech, Taipei, Taiwan). The result was expressed in mg Trolox/100 g.

The ability of osmotically dehydrated beetroot extracts to reduce ABTS-+ cation radicals was tested according to the methodology developed by Re et al. (1999) [84]. 0.192 g of ABTS was weighed, transferred to a volumetric flask (50 mL), and deionized water was added. Next, 0.0166 g of potassium persulfate was weighed out, transferred to a volumetric flask (25 mL), and refilled with deionized water. Solutions of ABTS and K_2_S_2_O_8_ (1.0:0.5 *v*/*v*) were mixed together and the obtained mixture was kept at room temperature (22 °C) in the dark for 16 h. The mixture was diluted with solvent to obtain an absorbance of 0.700 at λ = 734 nm. Then, 30 µL of extract was taken to which 3 mL of ABTS reagent was added and shaken on a vortex. After 6 min, the absorbance (λ = 734 nm) (Metertech SP 830) was measured. The result was expressed in mg Trolox/100 g.

Oxygen radical absorbance capacity of the extracts tested was carried out using the method developed by Ou et al. (2001) [85]. A series of solutions (2 nM fluorescein, 153 nM AAPH, 0.075 M phosphate buffer (pH 7.4)) were prepared. The beet extract was then dissolved in phosphate buffer. A reaction mixture was prepared by combining 0.04 µM disodium fluorescein and 0.075 M phosphate buffer. The extract was added to the prepared reaction mixture. The source of superoxide radicals in this assay was a 153 nM AAPH solution. The fluorescence of the samples was measured using a spectrofluorometer (F-2700, Hitachi, Tokyo, Japan) at an excitation wavelength of λ = 493 nm and an emission wavelength of λ = 515 nm. The result was expressed in mg Trolox/100 g.

### 3.7. Food Composition Analysis

An analysis of the content of basic nutrients (protein, fat, and fiber) in osmotically dehydrated beets was performed. For the determination of protein content, The Kjeldahl method with the use of a Kjeltec-2200 System (Foss Tecator, Hoganas, Sweden) was used according to AOAC [86]. Fat content was determined with the Soxhlet method using a Soxtec-HT6 System (Foss Tecator, Hoganas, Sweden) [87]. Total dietary fiber content was determined using the enzymatic-gravimetric Asp method using Fibertec (Foss Tecator, Hoganas, Sweden) [88].

### 3.8. Texture Profile Analysis (TPA)

Texture profile analysis (TPA) was performed using TA.XTplus Texture Analyzer (Stable Micro Systems Ltd., Godalming, UK) according to Kowalczewski et al., 2019 [89]. The following texture parameters were determined: hardness, adhesion, elasticity, cohesiveness, gumminess, chewiness, and resilience, as calculated by texturograph software (version 7.0.6.0 Stable Microsystems). Six replicates were analyzed for each sample. Texture profile analysis was performed on osmotically dehydrated, non-lyophilized samples.

### 3.9. Statistical Analysis

Statistically significant differences were determined using multivariate analysis of variance (ANOVA), followed by Tukey post hoc test (Statistica 13.1, StatSoft Inc., Kraków, Poland). The differences were considered statistically significant at *p* < 0.05. The experiments were conducted in triplicate.

## 4. Conclusions

The conducted study proved that osmotic dehydration can be used to enrich the plant matrix with nutrients. In addition, it was shown that the effectiveness of osmotic dehydration for food enrichment is significantly influenced by a number of process conditions, i.e., the type and concentration of the osmotically active substance, the chemical form, and concentration of potassium added to the hypertonic solution, as well as the time and temperature of the process. The highest increase in potassium content was observed in samples dehydrated in 50% solutions of erythritol and inulin with the addition of potassium chloride (5.0%) when the process lasted 180 min (respectively 5779.03 mg/100 g, 5457.28 mg/100 g).

In our previous studies, we demonstrated that osmotic dehydration is an effective method for enriching pumpkin flesh with calcium. We then used such a plant matrix in a rat model of postmenopausal osteoporosis and confirmed the positive health effects of calcium-enriched pumpkin. As a result, it can be concluded that the use of potassium-enriched beet can also find application in the prevention or support of civilization diseases, especially since potassium is a deficient component in the human diet. Therefore, the further direction of research should be to conduct an animal experiment to evaluate the health benefits of consuming potassium-enriched beets.

In addition to enriching foods with certain nutrients by osmotic dehydration per se, attention should also be paid to the practical aspect of shaping the sensory characteristics of foods, since the addition of mineral salts to a hypertonic solution can significantly modulate the taste of the final product. The next stage of the research will evaluate the suitability of using potassium-enriched beet pulp to develop a line of food products.

## Figures and Tables

**Table 1:** Potassium content of dehydrated beet (mg/100 g) before freeze-drying (stage 1).

Osmotically Active Substance	Potassium Gluconate	Potassium Citrate	Potassium Chloride
Water	744.32 ± 17.42 ^aA^	921.38 ± 75.02 ^aB^	1459.48 ± 39.1 ^aC^
IS25	1301.28 ± 20.95 ^bA^	3040.56 ± 84.1 ^bcB^	4058.56 ± 24.6 ^bC^
IS50	1913.75 ± 41.81 ^cA^	4515.05 ± 184.52 ^dB^	5059.92 ± 118.41 ^cC^
XS25	1584.18 ± 38.21 ^dA^	3123.46 ± 17.7 ^bB^	4691.13 ± 77.92 ^dC^
XS50	1731.22 ± 33.75 ^cdA^	3771.35 ± 80.53 ^fB^	5007.3 ± 116.63 ^cC^
ES25	1738.93 ± 71.18 ^cdA^	2819.39 ± 112.4 ^cB^	4679.74 ± 200.39 ^dC^
ES50	1934.5 ± 16.1 ^cA^	3155.13 ± 50.26 ^bB^	5165.99 ± 80.29 ^cC^
SS25	1537.4 ± 57.88 ^bdA^	2433.94 ± 80.72 ^eB^	4226.12 ± 75.8 ^beC^
SS50	1697.42 ± 50.09 ^cdA^	2900.45 ± 147.48 ^bcB^	4344.15 ± 67.23 ^eC^

a, b, c, d, e, f—different letters mean statistically significant differences (*p* < 0.05) between the osmotically active substances within the beetroot tested; A, B, C—different letters mean statistically significant differences (*p* < 0.05) between chemical forms of potassium within the beetroot tested; values are means of three determinations ± SD. IS25 and IS50—inulin solution (25 and 50%); XS25 and XS50—xylitol solution (25 and 50%); ES25 and ES50—erythritol solution (25 and 50%); SS25 and SS50—sucrose solution (25 and 50%).

**Table 2:** Results of multivariate analysis of variance for dehydrated beetroot samples (stage 1).

Combinations of Factors	*F*	*df*	*p*	*η* ^2^
Chemical form of potassium	6563.76	2.54	0.000	1.00
Osmotically active substance	746.54	8.54	0.000	0.99
Chemical form of potassium × Osmotically active substance	92.29	16.54	0.000	0.96

*F*—statistics analysis of variance, *df*—degrees of freedom, *p*—level of statistical significance, *η*^2^—effect strength.

**Table 3:** Potassium content of dehydrated beet (mg/100 g) after freeze-drying (stage 1).

Osmotically Active Substance	Potassium Gluconate	Potassium Citrate	Potassium Chloride
Water	10,951.72 ± 256.36 ^aA^	14,963.17 ± 1218.32 ^aB^	16,046.25 ± 510.19 ^aC^
IS25	2797.75 ± 45.03 ^bA^	11,517.43 ± 318.57 ^bB^	14,123.8 ± 85.62 ^bC^
IS50	5109.7 ± 111.63 ^cA^	7962.92 ± 325.43 ^cB^	10,825.12 ± 508.02 ^cC^
XS25	2566.37 ± 61.9 ^bA^	10,282.29 ± 58.26 ^dB^	14,279.79 ± 237.19 ^bC^
XS50	5314.83 ± 103.62 ^cA^	6105.81 ± 130.38 ^eB^	9934.72 ± 478.93 ^dC^
ES25	5848.98 ± 239.4 ^cA^	10,553.65 ± 420.76 ^bdB^	16,497.02 ± 834.88 ^aC^
ES50	3365.64 ± 28.01 ^bA^	7185.3 ± 114.45 ^cB^	11,019.06 ± 171.25 ^cC^
SS25	5880.58 ± 221.41 ^cA^	9502.44 ± 315.13 ^dB^	14,250.48 ± 255.6 ^bC^
SS50	3140.95 ± 92.69 ^bA^	4774.29 ± 242.76 ^fB^	11,753.89 ± 181.9 ^cC^

a, b, c, d, e, f—different letters mean statistically significant differences (*p* < 0.05) between the osmotically active substances within the beetroot tested; A, B, C—different letters mean statistically significant differences (*p* < 0.05) between chemical forms of potassium within the beetroot tested; values are means of three determinations ± SD. IS25 and IS50—inulin solution (25 and 50%); XS25 and XS50—xylitol solution (25 and 50%); ES25 and ES50—erythritol solution (25 and 50%); SS25 and SS50—sucrose solution (25 and 50%).

**Table 4:** Potassium content of dehydrated beet (mg/100 g) before freeze-drying (stage 2).

Time/Temperature		IS50		ES50	
		Potassium Chloride 2.5%	Potassium Chloride 5.0%	Potassium Chloride 2.5%	Potassium Chloride 5.0%
60 min	30 °C	2681.04 ± 37.6 ^aAxX^	4131.54 ± 63.62 ^aBxX^	2477.18 ± 68.22 ^bAxX^	3443.82 ± 65.22 ^bBxX^
	50 °C	3228.91 ± 55.56 ^aAxY^	4296.49 ± 87.46 ^aBxY^	2794.83 ± 66.56 ^bAxY^	3431.39 ± 95.41 ^bBxX^
120 min	30 °C	4107.25 ± 85.88 ^aAyX^	4952.74 ± 62.06 ^aByX^	3509.18 ± 58.73 ^bAyX^	5243.34 ± 76.11 ^bByX^
	50 °C	4101.51 ± 86.04 ^aAyX^	5086.54 ± 85.22 ^aByY^	4315.92 ± 49.87 ^bAyY^	5144.11 ± 63.87 ^aByX^
180 min	30 °C	4556.32 ± 50.16 ^aAzX^	5137.50 ± 127.20 ^aBzX^	3913.62 ± 58.06 ^bAzX^	5779.03 ± 60.55 ^bBzX^
	50 °C	4293.05 ± 21.07 ^aAzY^	5457.28 ± 117.25 ^aBzY^	4028.16 ± 77.87 ^bAzX^	5353.55 ± 92.24 ^aBzY^

a, b—different letters mean statistically significant differences (*p* < 0.05) between IS50 and ES50; A, B—different letters mean statistically significant differences (*p* < 0.05) between potassium chloride 2.5% and 5.0%; x, y, z—different letters mean statistically significant differences (*p* < 0.05) between samples dehydrated in different time conditions (60, 120, 180 min); X, Y—different letters mean statistically significant differences (*p* < 0.05) between samples dehydrated in different temperature conditions (30 and 50 °C). IS50—inulin solution (50%); ES—erythritol solution (50%).

**Table 5:** Results of multivariate analysis of variance for dehydrated beetroot samples (stage 2).

Combinations of Factors	*F*	*df*	*p*	*η* ^2^
Osmotically active substance	149.94	1.48	0.000	0.76
Potassium concentration	4025.16	1.48	0.000	0.99
Process time	2764.96	2.48	0.000	0.99
Process temperature	56.90	1.48	0.000	0.54
Osmotically active substance × Potassium concentration	35.45	1.48	0.000	0.43
Osmotically active substance × Process time	89.80	2.48	0.000	0.79
Osmotically active substance × Process temperature	0.85	1.48	0.361	0.02
Potassium concentration × Process time	11.49	2.48	0.000	0.32
Potassium concentration × Process temperature	45.91	1.48	0.000	0.49
Process time × Process temperature	31.60	2.48	0.000	0.57
Osmotically active substance × Potassium concentration × Process time	97.81	2.48	0.000	0.80
Osmotically active substance × Potassium concentration × Process temperature	99.59	1.48	0.000	0.68
Osmotically active substance × Process time × Process temperature	20.83	2.48	0.000	0.47
Potassium concentration × Process time × Process temperature	13.68	2.48	0.000	0.36
Osmotically active substance × Potassium concentration × Process time × Process time × Process temperature	28.87	2.48	0.000	0.55

*F*—statistics analysis of variance, *df*—degrees of freedom, *p*—level of statistical significance, *η*^2^—effect strength.

**Table 6:** Potassium content of dehydrated beet (mg/100 g) after freeze-drying (stage 2).

Time and Temperature		IS50		ES50	
		Potassium Chloride 2.5%	Potassium Chloride 5.0%	Potassium Chloride 2.5%	Potassium Chloride 5.0%
60 min	30 °C	5522.87 ± 77.45 ^aAxX^	8180.45 ± 125.97 ^aBxX^	4310.3 ± 118.71 ^bAxX^	6577.7 ± 124.56 ^bBxX^
	50 °C	6167.22 ± 106.12 ^aAxY^	8678.91 ± 176.66 ^aBxY^	3884.81 ± 92.52 ^bAxY^	7411.8 ± 206.08 ^bBxY^
120 min	30 °C	8255.57 ± 172.62 ^aAyX^	9806.42 ± 122.88 ^aByX^	5684.87 ± 95.14 ^bAyX^	8284.48 ± 120.25 ^bByX^
	50 °C	7546.78 ± 158.32 ^aAyY^	10,742.22 ± 169.58 ^aByY^	8329.72 ± 96.25 ^bAyY^	11,008.4 ± 136.68 ^bByY^
180 min	30 °C	6561.11 ± 72.23 ^aAzX^	9093.38 ± 225.15 ^aBzX^	7944.65 ± 117.87 ^bAzX^	12,078.18 ± 126.54 ^bBzX^
	50 °C	5280.45 ± 25.92 ^aAzY^	11,950.84 ± 230.99 ^aBzY^	5478.3 ± 105.9 ^aAzY^	10,546.49 ± 181.72 ^aBzY^

a, b—different letters mean statistically significant differences (*p* < 0.05) between IS50 and ES50; A, B—different letters mean statistically significant differences (*p* < 0.05) between potassium chloride 2.5% and 5.0%; x, y, z—different letters mean statistically significant differences (*p* < 0.05) between samples dehydrated in different time conditions (60, 120, 180 min); X, Y—different letters mean statistically significant differences (*p* < 0.05) between samples dehydrated in different temperature conditions (30 and 50 °C). IS50—inulin solution (50%); ES—erythritol solution (50%).

**Table 7:** The level of potassium absorption (%) in the analyzed samples of osmotically dehydrated beetroot.

Time and Temperature		IS50		ES50	
		Potassium Chloride 2.5%	Potassium Chloride 5.0%	Potassium Chloride 2.5%	Potassium Chloride 5.0%
60 min	30 °C	40.93 ± 0.57 ^aAxX^	31.54 ± 0.49 ^aBxX^	37.82 ± 1.04 ^bAxX^	26.29 ± 0.5 ^bBxX^
	50 °C	49.3 ± 0.85 ^aAxY^	32.8 ± 0.67 ^aBxX^	42.67 ± 1.02 ^bAxY^	26.19 ± 0.73 ^bBxX^
120 min	30 °C	62.71 ± 1.31 ^aAyX^	37.81 ± 0.47 ^aByX^	53.58 ± 0.9 ^bAyX^	40.03 ± 0.58 ^bByX^
	50 °C	62.62 ± 1.31 ^aAyX^	38.83 ± 0.65 ^aByX^	65.89 ± 0.76 ^bAyY^	39.27 ± 0.49 ^aByX^
180 min	30 °C	69.56 ± 0.77 ^aAzX^	39.22 ± 0.97 ^aBzX^	59.75 ± 0.89 ^bAzX^	44.11 ± 0.46 ^bBzX^
	50 °C	65.54 ± 0.32 ^aAzY^	41.66 ± 0.9 ^aBzY^	61.5 ± 1.19 ^bAzY^	40.87 ± 0.7 ^aByY^

a, b—different letters mean statistically significant differences (*p* < 0.05) between IS50 and ES50; A, B—different letters mean statistically significant differences (*p* < 0.05) between potassium chloride 2.5% and 5.0%; x, y, z—different letters mean statistically significant differences (*p* < 0.05) between samples dehydrated in different time conditions (60, 120, 180 min); X, Y—different letters mean statistically significant differences (*p* < 0.05) between samples dehydrated in different temperature conditions (30 and 50 °C). IS50—inulin solution (50%); ES—erythritol solution (50%).

**Table 8:** Antioxidant activity and proximate composition of osmotically dehydrated beetroot.

	Protein Content (g/100 g)	Fat Content (g/100 g)	Dietary Fiber Content (g/100 g)	DPPH (mg Trolox/100 g)	ABTS (mg Trolox/100 g)	ORAC (mg Trolox/100 g)
1A	1.24 ± 0.15 ^a^	0.12 ± 0.01 ^a^	1.76 ± 0.16 ^a^	108.35 ± 2.3 ^a^	36.85 ± 0.69 ^a^	242 ± 2.91 ^a^
2A	8.73 ± 0.29 ^b^	0.73 ± 0.05 ^b^	7.56 ± 0.16 ^c^	248.58 ± 5.27 ^b^	88.5 ± 1.65 ^b^	686.95 ± 8.23 ^b^
1B	1.32 ± 0.18 ^a^	0.12 ± 0.03 ^a^	1.74 ± 0.08 ^a^	75.65 ± 1.76 ^c^	26.54 ± 1.02 ^c^	174.99 ± 1.77 ^c^
2B	8.56 ± 0.45 ^b^	0.7 ± 0.06 ^b^	7.92 ± 0.1 ^c^	196.68 ± 4.56 ^d^	71.38 ± 2.74 ^d^	428.37 ± 4.33 ^d^
1C	1.32 ± 0.18 ^a^	0.19 ± 0.06 ^a^	1.62 ± 0.08 ^a^	109.77 ± 1.14 ^a^	27.17 ± 0.72 ^c^	176.04 ± 2.63 ^c^
2C	8.42 ± 0.08 ^b^	0.73 ± 0.08 ^b^	7.8 ± 0.14 ^c^	249.98 ± 2.59 ^b^	75.06 ± 20 ^d^	519.34 ± 7.75 ^e^
1D	1.1 ± 0.06 ^a^	0.09 ± 0.02 ^a^	1.69 ± 0.05 ^a^	81.35 ± 1.69 ^c^	21.42 ± 0.62 ^e^	176.54 ± 3.24 ^c^
2D	8.24 ± 0.22 ^b^	0.61 ± 0.08 ^b^	7.58 ± 0.06 ^c^	219.7 ± 4.57 ^e^	61.45 ± 1.79 ^f^	417.67 ± 7.67 ^d^
1E	1.72 ± 0.3 ^a^	0.27 ± 0.02 ^a^	2.37 ± 0.1 ^b^	242.07 ± 2.54 ^b^	63.24 ± 1.4 ^f^	1651.99 ± 17.40 ^f^
2F	10.83 ± 0.94 ^c^	1.2 ± 0.06 ^c^	15.3 ± 0.41 ^d^	741.59 ± 7.77 ^f^	174.51 ± 3.87 ^g^	1883.48 ± 105.33 ^g^

a, b, c, d, e, f, g—different letters mean statistically significant differences (*p* < 0.05). Sample 1A/2A—beet subjected to osmotic dehydration (50% inulin solution, 5.0% potassium chloride addition, 180 min, 30 °C, before and after freeze-drying; Sample 1B/2B—beet subjected to osmotic dehydration (50% inulin solution, 5.0% potassium chloride addition, 180 min, 50 °C, before and after freeze-drying; Sample 1C/2C—osmotically dehydrated beet (50% erythritol solution, 5.0% potassium chloride addition, 180 min, 50 °C, before and after freeze-drying; Sample 1D/2D—osmotically dehydrated beet (50% inulin solution, 5.0% potassium chloride addition, 180 min, 30 °C, before and after freeze-drying; Sample 1E—fresh beet; Sample F—freeze-dried beet.

**Table 9:** Texture profile analysis of beetroot pulp (TPA).

	Hardness [N]	Adhesiveness [N*s]	Springiness [%]	Cohesiveness	Gumminess	Chewiness	Resilience
S1	112.42 ± 5.62 ^a^	−0.18 ± 0.01 ^a^	0.44 ± 0.01 ^a^	0.27 ± 0.02 ^a^	3459.34 ± 169.13 ^a^	1673.2 ± 41.14 ^a^	0.19 ± 0.03 ^a^
S2	275.72 ± 4.06 ^b^	−0.07 ± 0.08 ^a^	0.57 ± 0.07 ^a^	0.48 ± 0.03 ^b^	16003.19 ± 362.28 ^b^	10731.83 ± 743.09 ^b^	0.33 ± 0.02 ^b^
S3	104.87 ± 9.22 ^a^	−0.06 ± 0.07 ^a^	0.46 ± 0.03 ^a^	0.3 ± 0.02 ^a^	3584.4 ± 634.89 ^a^	1857.56 ± 422.4 ^a^	0.21 ± 0.03 ^a^
S4	82.71 ± 3.72 ^c^	−0.21 ± 0.05 ^a^	0.61 ± 0.16 ^a^	0.58 ± 0.15 ^b^	4502.26 ± 691.99 ^a^	2572.09 ± 715.81 ^e^	0.42 ± 0.13 ^c^
S5	109.96 ± 2.04 ^a^	−0.15 ± 0.1 ^a^	0.49 ± 0.07 ^a^	0.34 ± 0.05 ^a^	3673.5 ± 241.41 ^a^	1772.29 ± 232.53 ^a^	0.25 ± 0.04 ^a^
S6	83.38 ± 4.76 ^c^	−0.06 ± 0.05 ^a^	0.44 ± 0.06 ^a^	0.29 ± 0.04 ^a^	2808.86 ± 195.62 ^a^	1413.94 ± 73.65 ^ac^	0.19 ± 0.03 ^a^
S7	28.64 ± 1.79 ^d^	−0.15 ± 0.04 ^a^	0.68 ± 0.03 ^b^	0.64 ± 0.07 ^b^	1740.31 ± 199.34 ^c^	1106.17 ± 93.23 ^cd^	0.37 ± 0.09 ^bc^

a, b, c, d, e—different letters mean statistically significant differences (*p* < 0.05). S1—fresh beet placed in water; S2—fresh beet; S3—beet subjected to osmotic dehydration (50% erythritol solution, without potassium chloride); S4—beet subjected to osmotic dehydration (50% inulin solution, without potassium chloride); S5—beet placed in a 5.0% potassium chloride solution; S6—beet subjected to osmotic dehydration (50% erythritol solution, 5.0% potassium chloride); S7—beet subjected to osmotic dehydration (50% inulin solution, 5.0% potassium chloride).

## Data Availability

The data presented in this study are available on request from the corresponding author.
